# Hypodontia as an extended phenotype in families with nonsyndromic oral clefts: findings from a 39-family clinical cohort

**DOI:** 10.1590/1678-7765-2026-0193

**Published:** 2026-09-11

**Authors:** Ana Laura Herrera Farha, Otávio Pagin, Izabel Maria Marchi de Carvalho, Ariadne Letra, Lucimara Teixeira das Neves

**Affiliations:** 1 Universidade de São Paulo Faculdade de Medicina de Bauru Programa de pós-graduação em Ciências da Reabilitação Bauru SP Brasil Universidade de São Paulo, Faculdade de Medicina de Bauru, Programa de pós-graduação em Ciências da Reabilitação, Bauru, SP, Brasil.; 2 Universidade de São Paulo Hospital de Clínicas de Bauru Seção de Diagnóstico Oral Bauru SP Brasil Universidade de São Paulo, Hospital de Clínicas de Bauru (Unidade HRAC), Seção de Diagnóstico Oral, Bauru, SP, Brasil.; 3 University of Pittsburgh School of Dental Medicine Department of Endodontics Pittsburgh Pennsylvania USA University of Pittsburgh School of Dental Medicine, Department of Endodontics and Department of Oral and Craniofacial Sciences, Center for Craniofacial and Dental Genetics, Pittsburgh, Pennsylvania, USA; 4 Universidade de São Paulo Faculdade de Odontologia de Bauru Departamento de Ciências Biológicas Bauru SP Brasil Universidade de São Paulo, Faculdade de Odontologia de Bauru, Departamento de Ciências Biológicas, Bauru, SP, Brasil.

**Keywords:** Cleft lip, Cleft palate, Hypodontia, Recurrence, Phenotype

## Abstract

Hypodontia is frequent in individuals with nonsyndromic oral clefts (NSOC) and has been proposed as a potential extended phenotype reflecting increased genetic susceptibility. However, hypodontia within the cleft area likely results from local anatomical disruption rather than a shared genetic etiology, limiting its usefulness in recurrence-risk assessment. Hypodontia outside the cleft area, though less prevalent, is more likely to reflect a genetically driven disturbance in odontogenesis and a more meaningful marker of familial susceptibility.

**Objective:**

In this observational cohort study, we investigated whether parents with NSOC and hypodontia outside the cleft area have a higher rate of cleft recurrence in their offspring compared with parents without hypodontia.

**Methodology:**

From a longitudinal cohort of 358 individuals with NSOC followed at the Hospital for Rehabilitation of Craniofacial Anomalies, Bauru, Brazil, from 2020 to 2022, participants who had biological children were interviewed regarding cleft recurrence and family history. Eligibility criteria included confirmed NSOC, at least one biological child, and availability of panoramic radiographs for hypodontia assessment.

**Results:**

Individuals were categorized as NSOC with hypodontia outside the cleft area (CH group) or isolated NSOC without hypodontia (IC group). Thirty-nine families met the inclusion criteria (9 CH; 30 IC). Cleft recurrence proportions were 22.2% in the CH group and 13.3% in the IC group. Descriptive and inferential analyses were performed; Fisher’s exact test was employed for comparisons. The estimated relative risk was 1.67 (95% confidence interval [CI]: 0.36–7.66; p=0.607). Although this difference was not statistically significant, the observed phenotypic pattern raises the hypothesis that hypodontia outside the cleft region may represent a potential marker of developmental susceptibility and etiological heterogeneity in NSOC.

**Conclusion:**

These findings support the value of comprehensive dental phenotyping in craniofacial research and warrant further investigation in larger family-based cohorts integrating phenotypic and genetic approaches.

## Introduction

Oral clefts (OC) are the most common craniofacial birth defects and result from developmental failure during embryogenesis.^1^ They can be classified as syndromic oral clefts, accounting for approximately 30% of cases, or nonsyndromic oral clefts (NSOC), which represent nearly 70%.^2^ NSOC is a complex trait with multifactorial etiology involving both genetic and environmental determinants, resulting in a complex inheritance pattern.^3^ Genetically, NSOC may occur sporadically (approximately 80% of cases), suggesting de novo events, or through familial aggregation.^4^ Familial recurrence and consanguinity have been extensively investigated,^5,6^ and a positive family history remains one of the strongest risk factors for NSOC.^6-9^ Recurrence risk decreases with kinship distance due to reduced allele sharing,^10^ supporting a multifactorial threshold model in which shared genetic liability influences the probability of recurrence.^7^ Sibling recurrence is approximately 4%, compared with 0.2% among siblings of unaffected infants.^11,12^

Because NSOC has a strong genetic component, it may also affect other structures derived from the ectomesenchyme, including the dental lamina. Consequently, dental anomalies are highly prevalent in individuals with oral clefts,^13^ with hypodontia being one of the most common findings.^14,15^ OC and hypodontia are among the most frequent developmental orofacial defects, and multiple studies have reported associations between the two anomalies.^15,16^ Both conditions also show familial recurrence, suggesting a potential shared genetic susceptibility.^17,18^ In addition, unaffected relatives of individuals with OC show higher frequencies of hypodontia, reinforcing this connection.^13^ Genetic evidence further supports this relationship: several variants associated with hypodontia—such as *MSX1, PAX9, CDH1, IRF6, TBX22, TGFA, TGFB3*, and *WNT* pathway genes—have also been implicated in NSOC independently or in combination.^19-25^

In this context, an important distinction must be considered when interpreting dental agenesis in individuals with NSOC. Although hypodontia is frequently observed in this population, its occurrence is not uniform across the dentition. Tooth absence is particularly common in regions adjacent to the cleft site, where local developmental disturbances—such as disruption of the dental lamina or surgical interventions—may contribute to agenesis. In these cases, missing teeth may reflect secondary, mechanically induced effects rather than underlying genetic susceptibility.

In contrast, patterns of agenesis that extend beyond the cleft region are less likely to be explained solely by local factors and may instead indicate involvement of shared developmental pathways. This distinction highlights the importance of not only identifying the presence of hypodontia but also considering its anatomical distribution and pattern when interpreting its role as a potential marker of genetic susceptibility in NSOC.

Although the distribution patterns of hypodontia in NSOC have been widely described, their clinical application as markers of recurrence risk remains limited. In particular, dental anomalies—especially hypodontia outside the cleft area—have rarely been incorporated into recurrence risk assessments. To date, no study has evaluated whether the concomitant presence of NSOC and hypodontia outside the cleft area in a parent increases the recurrence of clefts in their offspring. Therefore, this study tested the hypothesis that parents with NSOC and hypodontia outside the cleft area have a higher rate of oral cleft recurrence in their children than parents with NSOC without hypodontia. By comparing familial recurrence patterns between these groups, this study sought to assess whether hypodontia outside the cleft area may serve as an additional predictor of recurrence risk, with direct clinical relevance for genetic counseling and public health strategies aimed at prevention.

## Methodology

### STUDY DESIGN AND ETHICAL APPROVAL

This observational cohort study was conducted with individuals enrolled for treatment at the Hospital for Rehabilitation of Craniofacial Anomalies, Brazil. The study was conducted in accordance with ethical principles and was approved by the Research Ethics Committee (CAAE: 55667622.5.0000.5441). Participants were contacted via email or phone for eligibility screening and invited to participate between 2020 and 2022.

### STUDY POPULATION AND ELIGIBILITY CRITERIA

A total of 358 individuals were initially screened. Families were included if the proband had a confirmed diagnosis of NSOC, had at least one biological child, and had available dental records, including panoramic radiographs obtained at ≥9 years of age to allow assessment of hypodontia.

Exclusion criteria included individuals with suspected syndromes or structural anomalies, those in whom hypodontia status or the presence of permanent teeth could not be reliably confirmed, and those without biological children.

### ASSESSMENT OF HYPODONTIA

Dental agenesis was assessed through comprehensive evaluation of archived imaging, including periapical and panoramic radiographs, with emphasis on panoramic images.

The diagnosis was based on the absence of any radiographic evidence of mineralized dental tissue (enamel or dentin) in the region of the corresponding tooth. In cases where multiple imaging exams were available, all records were reviewed to confirm the diagnosis. A thorough review of dental records was also performed to ensure that missing teeth were not due to other clinical reasons. All radiographic evaluations were conducted by a single trained examiner.

The number of missing teeth per individual was recorded for descriptive purposes. Teeth considered to be within or directly associated with the cleft region were defined as the maxillary lateral incisors on the affected side in cases of unilateral clefts and on both sides in cases of bilateral clefts. Only maxillary teeth were considered in this definition, as mandibular teeth are not anatomically involved in the cleft area. All other teeth, including maxillary posterior teeth and all mandibular teeth, were classified as outside the cleft region. This approach was adopted to ensure that agenesis patterns reflect systemic developmental alterations rather than secondary effects associated with the cleft. Third molars were also excluded from all analyses.

### GROUP ALLOCATION

Participants were allocated into two groups based on the presence or absence of hypodontia: CH group (NSOC with hypodontia outside the cleft area) and IC group (isolated NSOC without hypodontia).

Individuals were classified as tooth agenesis-positive (CH group) if they presented agenesis of one or more permanent teeth located outside the cleft region, excluding third molars. This classification included individuals with agenesis exclusively outside the cleft area as well as those presenting combined agenesis affecting both cleft and non-cleft regions. Individuals with agenesis restricted to the cleft region were excluded.

Participants who had NSOC and all permanent teeth, excluding third molars, were included in the IC group. Exceptionally, for the IC group only, participants without archived radiographs were included if archived clinical photographs allowed confirmation of the presence of all teeth.

### CLEFT CLASSIFICATION

Clefts were classified into three main types: isolated cleft lip, cleft lip and palate, and isolated cleft palate. Laterality was recorded for isolated cleft lip and cleft lip and palate.

### DATA COLLECTION AND PROCEDURES

Participants who met the eligibility criteria and agreed to participate were interviewed. The informed consent form was provided and signed via Google Forms. Interviews were conducted either virtually or in person, when the volunteer already had an appointment previously scheduled at our service.

A structured interview guide with open-ended questions (Supplementary Material S1) was used to collect information on family structure, number of children, sex, parental age at conception, presence of NSOC in offspring, cleft type, consanguinity, and family history of OC or other anomalies.

The interview also addressed questions about the gestational history of the children born with OC. The participants were questioned about active or passive smoking, maternal and paternal alcohol intake during the periconceptional period, nutritional intake, use of prescribed medications, use of recreational or illicit drugs, and maternal diseases.

Clinical and demographic data were supplemented with information obtained from archived medical records.

### STATISTICAL ANALYSIS

The primary unit of analysis for recurrence was the family, with each index case representing one family. Recurrence was defined dichotomously based on the presence of at least one affected offspring, regardless of the total number of children. Thus, participants were classified as having recurrence if any of their children were affected and as having no recurrence if none of their children were affected. Multiple affected offspring within the same family were not treated as independent observations but were incorporated into a single family-level outcome variable. Therefore, recurrence estimates reflect the proportion of families with at least one affected offspring rather than the proportion of affected offspring.

No formal sample size calculation was performed *a priori*, as this was an exploratory study investigating a rare phenotypic subgroup.

Statistical analyses were conducted using both descriptive and inferential approaches. Categorical variables were summarized as absolute frequencies and percentages, with 95% confidence intervals (CIs) for proportions estimated using the Wilson score method, which is recommended when sample sizes are small or observed proportions are extreme. Fisher’s exact test was used to compare cleft recurrence rates between groups (CH *vs*. IC) and between sexes. The relative risk of cleft recurrence in the CH group compared with the IC group was calculated with its 95% confidence interval using the standard logarithmic method for 2×2 contingency tables. Although inferential measures were computed, all results were interpreted with appropriate caution given the exploratory nature of the study and its limited statistical power. The statistical analysis was performed using R statistical software (version 4.5.2; R Foundation for Statistical Computing, Vienna, Austria). The significance level was set at p<0.05 for all analyses.

## Results

Of the 358 individuals with NSOC in the source cohort, 319 were excluded for reasons described in **Figure 1**. Thus, 39 met all inclusion criteria and were enrolled in the study: nine in the CH group and 30 in the IC group. The distribution of OC types in both groups is shown in **Table 1**.

**Figure 1:**
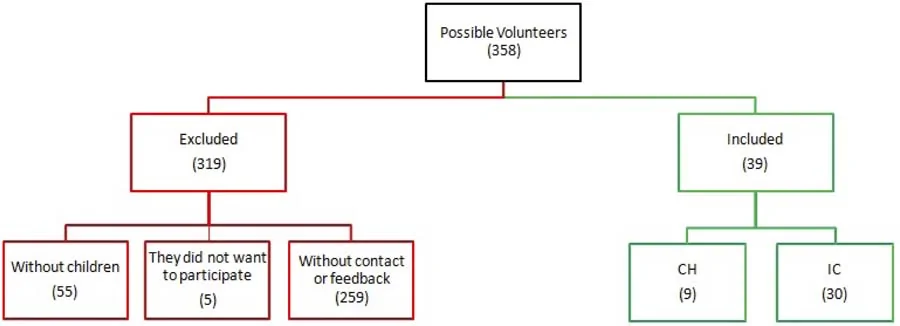
Screened participants for the study: distribution of included participants and reasons for exclusion.

**Table 1:** Distribution of cleft types in the CH and IC groups.

Cleft type	CH (n=9)	IC (n=30)
CL (left)	-	2
CL (right)	-	1
CLA (left)	-	2
CLA (right)	-	1
CLP (left)	4	9
CLP (right)	2	2
CLP (bilateral)	1	7
CLA (bilateral)	1	1
CLP (left) + CL (right)	1	-
CLP (right) + CL (left)	-	2
Isolated CP	-	1
Combined clefts (CLA left + CP)	-	2

Abbreviations: CL: cleft lip; CLA: cleft lip and alveolus; CLP: cleft lip and palate; CP: cleft palate; CH: nonsyndromic oral cleft (NSOC) with hypodontia outside the cleft area; IC: isolated NSOC without hypodontia

Among the nine participants with hypodontia outside the cleft area (CH group), three were male (33.3%) and six were female (66.7%). A detailed characterization of dental agenesis in the CH group is presented in **Table 2**.

**Table 2:** Detailed profile of dental agenesis outside the cleft area in the CH group.

ID	Sex	Cleft Type	Hypodontia outside the cleft area *	Hypodontia within the cleft area *	Number of Missing Teeth	Pattern (outside the cleft area)
1	F	CLP (left)	25	22	2	Unilateral
2	M	CL (right) + CLP (left)	15	12, 22	3	Unilateral
3	F	CLP (left)	15, 25, 35	22	4	Bilateral, asymmetrical
4	F	CLP (bilateral)	15	12	2	Unilateral
5	F	CLP (left)	45	22	2	Unilateral
6	F	CLP (right)	15, 25	-	2	Bilateral, symmetrical
7	M	CLA (bilateral)	15, 35	12, 22	4	Bilateral, asymmetrical
8	F	CLP (left)	15, 25, 35, 45	-	4	Bilateral, symmetrical
9	M	CLP (right)	25	12	2	Unilateral

* FDI: Fédération Dentaire Internationale notation. Only teeth located outside the cleft region were considered in this analysis

Abbreviations: F: female; M: male; CL: cleft lip; CLP: cleft lip and palate

All nine individuals in the CH group exhibited at least one missing tooth outside the cleft region, with the total number of absent teeth ranging from two to four. The most frequently affected teeth were the maxillary second premolars (FDI 15 and 25). Several individuals presented bilateral and, in some cases, symmetrical patterns of tooth absence, while others exhibited unilateral or asymmetrical distributions. In three cases, agenesis affected both maxillary and mandibular arches.

In the IC group, 10 participants were male (33.3%) and 20 were female (66.7%) **(Figure 2**). A positive family history of oral clefts was reported by 44.4% of the CH group (n=4) and 43.3% of the IC group (n=13), with no significant difference between them (p=1.0). In all cases, the affected relative was a distant relative on the cleft-affected parent’s side of the family; unaffected parents reported no family history of oral clefts. There were no cases of parental consanguinity in the groups.

**Figure 2:**
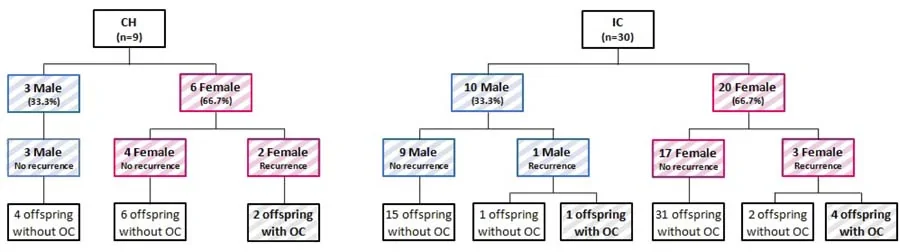
Distribution of cleft recurrence, sex of the parent, and presence or absence of oral cleft in the offspring in the CH and IC groups. Blue boxes represent male individuals, pink boxes represent female individuals, and hatched boxes indicate the presence of oral cleft. The presence of an oral cleft in the offspring is also bold. Abbreviations: CH: nonsyndromic oral cleft (NSOC) with hypodontia outside the cleft area; IC: isolated NSOC without hypodontia

At the family level, cleft recurrence in offspring was observed in 2 of 9 families in the CH group (22.2%; 95% CI: 6.3%–54.7%) and in 4 of 30 families in the IC group (13.3%; 95% CI: 5.3%–29.7%). The relative risk of cleft recurrence comparing CH parents with IC parents was 1.67 (95% Confidence Interval: 0.36–7.66; p=0.607, Fisher’s exact test).

When recurrence was evaluated by sex within each group, 33.3% of women in the CH group had children with oral clefts (n=2/6), whereas none of the male participants had affected offspring. In the IC group, recurrence occurred in 15% of women (3/20) and 10% of men (1/10). Despite these numerical differences, no sex-related differences were statistically significant (CH: p=0.50; IC: p=1.0, both Fisher’s exact test).

At the offspring level, 12 children were born to CH participants, of whom two (16.7%) had OC; these cases occurred in offspring of different parents (Figure 2). In the IC group, 54 children were born, and five (9.3%) presented OC; these five belonged to four families, with two siblings among them (**Figure 2**). All children born with OC are currently being followed at our institution.

When offspring outcomes were analyzed by parental sex, 25% (2/8) of the children of female CH participants had OC, whereas none of the four children of male CH participants were affected. In the IC group, the proportion of offspring with OC was 10.8% (4/37) among children of female participants and 5.8% (1/17) among children of male participants. However, these differences were not statistically significant (CH: p=0.515; IC: p=1.0). When comparing CH and IC groups separately by sex, no significant differences were found (women: p=0.286; men: p=1.0). Detailed recurrence data, including pregnancy outcomes, are presented in **Table 3**.

**Table 3:** Clinical, familial, and gestational characteristics of NSOC recurrence cases in the CH and IC groups.

ID	Group	Sex	Cleft type	Family history	Hypodontia outside cleft	Hypodontia within cleft area	Offspring outcomes *	Relevant exposures
3	CH	F	CLP (left)	Yes	3 teeth	1 tooth	1 daughter with CLP (bilateral)	Alcohol and cannabis use during pregnancy
4	CH	F	CLP (bilateral)	Yes	1 tooth	1 tooth	1 unaffected daughter; 1 son with CLP (bilateral)	None
10	IC	F	CL (left) cicatricial	Yes	-	-	1 daughter with CLA (left); 2 unaffected children	None
11	IC	F	CLP (right)	Yes	-	-	1 son with CLP (bilateral)	Prior miscarriage (12 weeks)
12	IC	F	CLP (bilateral)	No	-	-	1 son with CL (left); 1 son with CLP (bilateral)	Smoking, antacids, antiemetics
13	IC	M	CLP (bilateral)	Yes	-	-	1 unaffected son; 1 son with CLA (left) + CP	Paternal alcohol use; maternal kidney condition

* Children in order of birth

Abbreviations: F: female; M: male; CL: cleft lip; CLA: cleft lip and alveolus; CLP: cleft lip and palate; CP: cleft palate; CH: Oral Cleft associated with Hypodontia; IC: Isolated Cleft

## Discussion

OC have substantial functional, aesthetic, and psychosocial consequences and require long-term multidisciplinary care. Understanding their etiology is therefore essential for improving prevention strategies and refining genetic counseling. In NSOC, although the genetic component is recognized, this aspect still represents a challenge, and important gaps remain in identifying clinical markers that may help predict recurrence risk.

In this context, it has been proposed that expanding the spectrum of phenotypic features considered part of the affected phenotype may increase the power to detect susceptibility factors. Accumulated evidence suggests that NSOC may not constitute a strictly dichotomous condition but rather a spectrum of developmental anomalies with variable expressivity. This concept of an expanded phenotype encompasses not only overt clinical forms of clefting but also subclinical manifestations, such as labial microforms, defects in the orbicularis oris muscle, and dental anomalies.^26^ The incorporation of these subphenotypes has contributed to a more comprehensive understanding of the genetic architecture of NSOC and its clinical variability.

Within the landscape of dental anomalies, hypodontia has been recognized as a potential phenotypic marker of susceptibility to NSOC, as its increased prevalence among both affected individuals and their unaffected first-degree relatives points to a common underlying genetic basis.^13,27,28^

A major challenge in investigating hypodontia as an extended phenotype of NSOC is distinguishing tooth agenesis that occurs within the cleft region from agenesis affecting teeth outside the cleft area. Although this distinction is critical for etiological interpretation, it has not been consistently addressed in the literature. Missing teeth located within the cleft region are highly prevalent and may arise, at least in part, from local developmental perturbations directly related to cleft formation, including disruption of odontogenesis in the affected craniofacial structures. Consequently, their inclusion may introduce phenotypic heterogeneity by conflating localized developmental effects with broader biological mechanisms underlying susceptibility to dental anomalies. In contrast, agenesis occurring outside the cleft region is less likely to be attributable to the local consequences of the cleft itself and may therefore more accurately capture systemic developmental and genetic factors shared between hypodontia and NSOC. To minimize this potential source of misclassification, we adopted a stringent phenotypic definition that included only individuals presenting tooth agenesis outside the cleft region, regardless of whether agenesis was also present within the cleft area. Although this strategy reduced the sample size and, consequently, statistical power, it enhanced phenotypic specificity and strengthened the biological relevance of the analyzed trait.

Beyond the presence of hypodontia itself, the pattern of tooth agenesis may provide important insights into the developmental mechanisms underlying this phenotype. Among affected individuals, agenesis predominantly involved premolars and, in some cases, extended across multiple quadrants or presented bilaterally. These characteristics are particularly relevant because the distribution of missing teeth is unlikely to be biologically neutral. Bilateral and more extensive forms of agenesis have been proposed to reflect a stronger developmental perturbation than isolated tooth absence, suggesting the involvement of mechanisms that act broadly during odontogenesis rather than localized events affecting individual tooth germs. Likewise, the frequent involvement of premolars is noteworthy, as these teeth are among the most affected in genetically mediated forms of hypodontia and are especially sensitive to alterations in the regulatory pathways governing tooth initiation and morphogenesis. The prevalence of second premolar agenesis observed in this study is consistent with previous literature, in which this pattern is widely described as one of the most frequent in cases of hypodontia.^29^

The biological relevance of these patterns is supported by evidence that tooth development and craniofacial morphogenesis share a partially overlapping genetic architecture. Genes such as *MSX1*, *PAX9*, *IRF6*, and components of the WNT signaling pathway play key roles in both processes, and variants in these genes have been associated with dental agenesis, NSOC, or both.^19-25^ Within this context, the predominance of premolar agenesis and the occurrence of bilateral or multi-quadrant involvement are compatible with the hypothesis that hypodontia outside the cleft region represents a manifestation of broader developmental susceptibility rather than a consequence of local disruption associated with cleft formation.^30^ Although the present findings do not provide direct evidence of a shared genetic etiology, the observed phenotypic patterns are consistent with current developmental models linking odontogenesis and craniofacial development.

If hypodontia outside the cleft region reflects an underlying developmental susceptibility to NSOC, one might expect its presence to be associated with a higher familial recurrence rate. To explore this possibility, recurrence patterns were compared between parents with and without hypodontia outside the cleft area. While the estimated relative risk was greater than unity (relative risk [RR] = 1.67), the association was not statistically significant and was characterized by a wide confidence interval (0.36–7.66), precluding firm conclusions. The small number of individuals in the CH group (n=9) substantially limited statistical precision, and larger studies will be required to determine whether hypodontia has value as a marker of familial recurrence risk.

In the present sample, NSOC recurrence was observed in 22.2% of CH families and in 13.3% of IC families. While these proportions should not be interpreted as precise estimates of recurrence risk, the observed trend is consistent with the hypothesis that hypodontia may identify a subgroup of families with increased developmental susceptibility. The primary value of this analysis therefore lies not in estimating recurrence risk itself but in exploring hypodontia outside the cleft region as a potential indicator of etiological heterogeneity within NSOC.

The recurrence proportions observed in this study were higher than those reported in population-based investigations, where recurrence among first-degree relatives has generally ranged from 2.2% to 7.6%.^11,31^ This discrepancy is likely attributable to differences in ascertainment and study design, given that our cohort was recruited from a tertiary craniofacial referral center and specifically evaluated recurrence among affected parents and their offspring. Consequently, direct comparisons between studies are limited. Although the present findings remain preliminary, they support further investigation of hypodontia as a potential marker of shared developmental and genetic susceptibility in larger family-based cohorts.

The variability in maternal and environmental factors observed among recurrence cases further reflects the multifactorial etiology of NSOC. Accordingly, the phenotypic patterns identified in this study should be interpreted within a broader framework in which genetic susceptibility interacts with environmental and developmental factors. This complexity likely extends to hypodontia, for which both genetic and non-genetic influences have been implicated, reinforcing the need for integrative approaches when investigating the biological basis of these traits.

The limitations of this study should be considered when interpreting the findings. Recruitment from a tertiary craniofacial referral center may have enriched the sample for familial cases and more complex phenotypes, potentially influencing recurrence estimates and limiting generalizability. In addition, the requirement that participants had biological offspring substantially reduced the eligible sample. Consistent with previous reports, many adults with NSOC indicated that concerns about recurrence influenced their reproductive decisions,^32^ further constraining recruitment.

Another limitation of this study is the lack of stratification by cleft type (cleft lip, cleft lip and palate, or isolated cleft palate). Given the relatively small sample size, further subgroup analyses were not feasible without substantially reducing statistical power. Consequently, it was not possible to determine whether the observed associations are consistent across cleft subtypes or driven by a specific subgroup. This limitation should be considered when interpreting the results, and future studies with larger, stratified samples are needed to clarify the potential influence of cleft type on the relationship between hypodontia and recurrence risk.

Despite these limitations, the study benefited from rigorous phenotypic characterization and careful assessment of dental anomalies outside the cleft region. The predominance of premolar agenesis and the frequent occurrence of bilateral or multi-quadrant involvement reinforce the biological plausibility of hypodontia as a manifestation of broader developmental susceptibility rather than a localized consequence of cleft formation. These observations are consistent with current models proposing shared developmental pathways between odontogenesis and craniofacial morphogenesis and underscore the value of detailed dental phenotyping in studies of NSOC.

## Conclusion

In conclusion, this study provides exploratory evidence that hypodontia outside the cleft region may reflect broader developmental susceptibility in individuals with NSOC. Although the observed difference in recurrence patterns did not reach statistical significance, the phenotypic distribution of dental agenesis raises the hypothesis that hypodontia may represent a potential marker of etiological heterogeneity within NSOC. These findings should be considered hypothesis-generating and warrant further investigation in larger family-based studies incorporating consistent phenotypic criteria and complementary genetic approaches.

The present findings also emphasize the value of detailed dental phenotyping in the clinical characterization of individuals with NSOC. As evidence from future studies accumulates, systematic assessment of dental anomalies may contribute to a better understanding of the developmental and genetic complexity underlying craniofacial conditions and inform future approaches to risk assessment.

Fundin: This study was financed in part by the Coordination for the Improvement of Higher Education Personnel (CAPES) – Finance Code 001 and by the São Paulo Research Foundation (FAPESP), grant no. [2024/18278-0 (LTN) and 2024/18301-2 (ALHF)].
